# Is T2 mapping reliable in evaluation of native and repair cartilage tissue of the knee?

**DOI:** 10.1186/s40634-021-00350-1

**Published:** 2021-04-28

**Authors:** Hasan Banitalebi, Christian Owesen, Asbjørn Årøen, Hang Thi Tran, Tor Åge Myklebust, Per-Henrik Randsborg

**Affiliations:** 1 Department of Diagnostic Imaging, Akershus University Hospital, 1478 Lørenskog, Norway; 2 Institute of Clinical Medicine, University of Oslo, Oslo, Norway; 3 Department of Orthopaedic Surgery, Akershus University Hospital, 1478 Lørenskog, Norway; 4 Oslo Sports Trauma Research Centre, Oslo, Norway; 5 Department of Research and Innovation, Møre and Romsdal Hospital Trust, Ålesund, Norway

**Keywords:** T2 mapping, MRI, Articular cartilage, Reliability, Cartilage repair, Autologous chondrocyte implantation

## Abstract

**Purpose:**

To evaluate the effect of imaging plane and experience of observers on the reliability of T2 mapping of native and repair cartilage tissue of the knee.

**Methods:**

Fifteen consecutive patients from two randomised controlled trials (RCTs) were included in this cross-sectional study. Patients with an isolated knee cartilage lesion were randomised to receive either debridement or microfracture (RCT 1) or debridement or autologous chondrocyte implantation (RCT 2). T2 mapping was performed in coronal and sagittal planes two years postoperatively. A musculoskeletal radiologist, a resident of radiology and two orthopaedic surgeons measured the T2 values independently. Intraclass Correlation Coefficient (ICC) with 95% Confidence Intervals was used to calculate the inter- and intraobserver agreement.

**Results:**

Mean age for the patients was 36.8 ± 11 years, 8 (53%) were men. The overall interobserver agreement varied from poor to good with ICCs in the range of 0.27– 0.76 for native cartilage and 0.00 – 0.90 for repair tissue. The lowest agreement was achieved for evaluations of repair cartilage tissue. The estimated ICCs suggested higher inter- and intraobserver agreement for radiologists. On medial femoral condyles, T2 values were higher for native cartilage on coronal images (*p* < 0.001) and for repair tissue on sagittal images (*p* < 0.001).

**Conclusions:**

The reliability of T2 mapping of articular cartilage is influenced by the imaging plane and the experience of the observers. This influence may be more profound for repair cartilage tissue. This is important to consider when using T2 mapping to measure outcomes after cartilage repair surgery.

**Trial registration:**

ClinicalTrials.gov, NCT02637505 and NCT02636881, registered December 2015.

**Level of evidence:**

II, based on prospective data from two RCTs.

**Supplementary Information:**

The online version contains supplementary material available at 10.1186/s40634-021-00350-1.

## Introduction

Measurement of T2 relaxation time (T2 mapping) by Magnetic Resonance Imaging (MRI) is an effective method to detect early degenerative changes in the hyaline cartilage [3, 5, 20]. The extracellular matrix of the cartilage is composed of three components: proteoglycan, collagen type II fibres and water. In normal cartilage, the proteoglycan molecule with its negatively charged glycosaminoglycan chains attracts water into the extracellular matrix. T2 mapping is capable of detecting the changes in the water content, as well as the content and the orientation of the collagen fibres in the cartilage [11, 12, 31, 32]. Studies suggest an association between the T2 values and the glycosaminoglycan content of cartilage [12, 31]. T2 mapping has been used to evaluate the postoperative results of surgical repair of cartilage damages with different repair techniques [14, 19, 26, 29, 30].

Publications have reported generally good reliability and reproducibility for measurements of T2 values of articular cartilage [4, 15, 18]. However, most reliability studies have used only one observer, while others lack information regarding blinding, medical specialty and the range of experience of the observers with T2 mapping [15, 6]. Further, the possible influence of the imaging plane on the T2 values and the reliability of these measurements has rarely been examined. There is therefore a need to evaluate the reliability of T2 mapping of cartilage repair tissue in more than one plane and by several observers with different levels of experience. Studies performed on laminar analysis of the cartilage (dividing the cartilage into two or three layers from deep to superficial) have shown mostly good inter- and intraobserver reliability [10, 9, 17, 18, 23], although a tendency towards lower agreement values compared to single-layer analyses has been reported [15].

The Norwegian Cartilage Project (NCP) [1, 22] is an ongoing multicentre study that compares the clinical and radiological outcomes after different surgical repair techniques, aiming to improve the treatment of injured articular cartilage of the knee. We hypothesised that the reliability of T2 measurements of articular cartilage is influenced by experience of the observers and the plane in which the images are acquired. Thus, the aim of the present study was to examine the inter- and intraobserver reliability of T2 mapping MRI of native cartilage and repair cartilage tissue of the knee with image acquisition in sagittal and coronal planes.

## Methods

This study has received ethics approval from the Regional Committee for Medical and Health Research Ethics, North-Norway (approval numbers 2015/2200 and 2015/2202) and the Institutional Data Protection Officer (reference number 2017_187). All patients provided written informed consent before inclusion. All aspects of the study were in accordance with the Declaration of Helsinki.

### Sample size

A power analysis at the 5% significance level and power of 80% was performed according to the method of Walter et al. [27]. With a sample size of 15, the study has a power to detect a difference in ICC of 0.3 when testing the null hypothesis that ICC is larger than 0.4.

### Patients

The NCP consists of two randomised controlled trials (RCTs). Both trials include patients aged 18–50 years with a single symptomatic cartilage lesion on the femoral condyles or the trochlea. RCT 1 [1] compares microfracture to arthroscopic debridement for cartilage lesions smaller than two cm^2^. RCT 2 [2] compares autologous chondrocyte implantation (ACI) to arthroscopic debridement for lesions larger than 2 cm^2^. The first 15 consecutive patients (convenience sampling) from these two trials (eight patients from RCT 1 and seven patients from RCT 2) were included in the current cross-sectional study, with the same inclusion and exclusion criteria as the main trials (Table 1).

**Table 1:** Inclusion and exclusion criteria

Inclusion	Exclusion
• Men and women between 18 and 50 years • Single symptomatic cartilage defect on medial or lateral femoral condyle, or on trochlea • Lesion size ≤ 2 cm^2^ for RCT 1 and > 2 cm^2^ for RCT 2 • Stable ligaments • Acceptable range of motion (5–105°)	**•** Osteoarthritis, rheumatoid or other systemic joint diseases **•** Malalignment > 5° measured on HKA images **•** Obesity (Body mass index ≥ 30) **•** Comorbidities that might influence the surgery or the rehabilitation **•** Inability to complete questionnaires or rehabilitation **•** Alcohol or drug abuse **•** Previous surgery on chondral defect (except surgery for OCD) **•** Pregnancy

Inclusion and exclusion criteria for the Norwegian Cartilage Project and the current study

*OCD* Osteochondritis Dissecans, *HKA* Hip-Knee-Ankle, *RCT 1* Randomised Controlled Trial 1: Debridement vs. Microfracture, *RCT 2* Randomised Controlled Trial 2: Debridement vs. ACI (Autologous Chondrocyte Implantation)

**Fig. 1:**
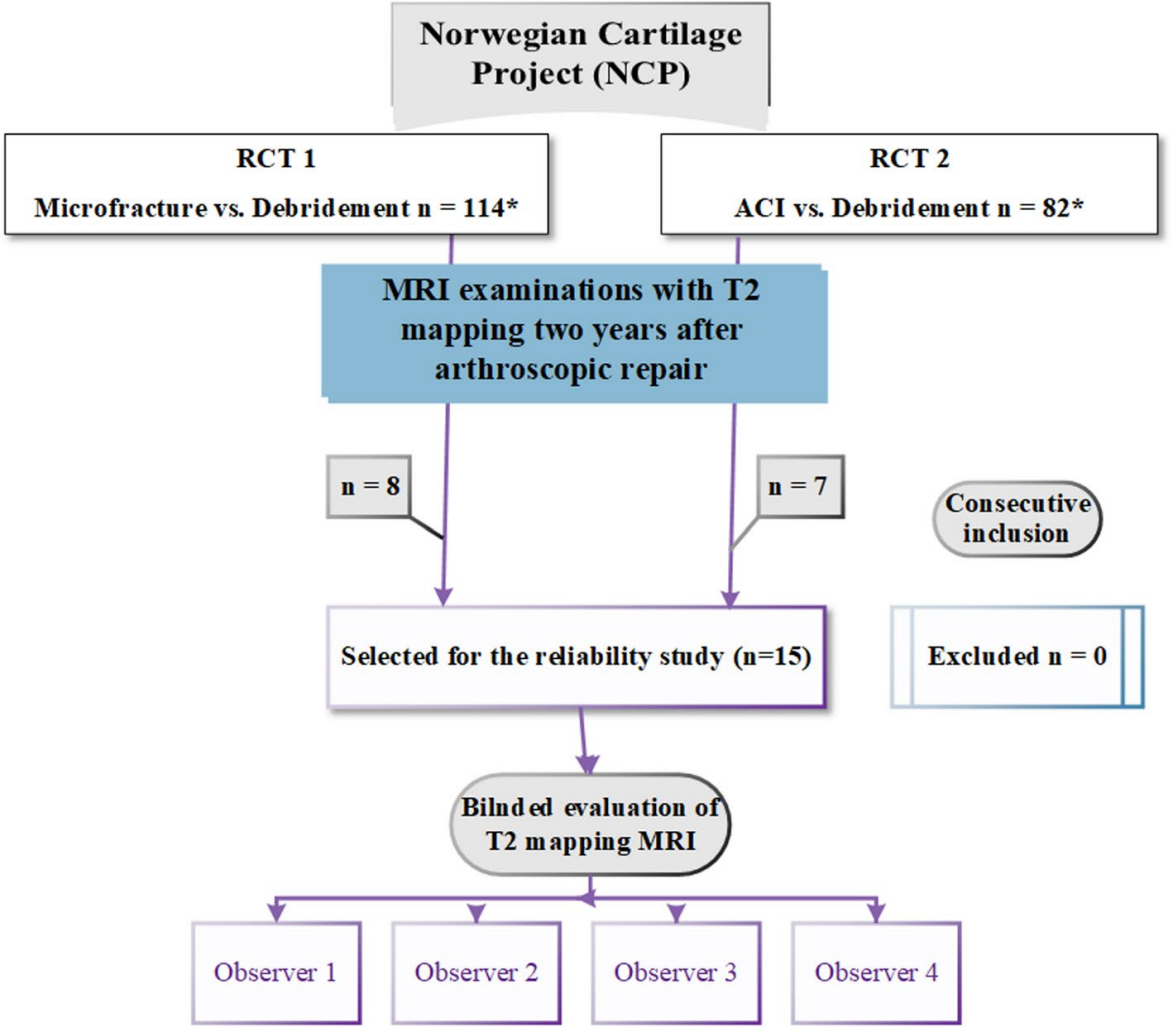
Flow diagram for the inclusion of the patients in the current reliability study from the two ongoing Randomised Controlled Trials of the Norwegian Cartilage Project. ACI: Autologous Chondrocyte Implantation. *Ongoing inclusion

Eight of the 15 included patients were men (53%). The mean age was 36.8 ± 11 years (33.8 for men and 41.5 for women). There were no exclusions. The patients were examined with T2 mapping MRI two years postoperatively (mean time 733 ± 22 days). The mean size of the cartilage lesions as measured arthroscopically was 3.4 ± 2.6 cm^2^. Demographic characteristics of the patients are presented in Table 2. The flow diagram for the inclusion of the patients is demonstrated in Fig. 1.

**Table 2:** Demographic characteristics and frequency distribution of the patients and treated lesions

Patient No	Age (years)	Sex	BMI	Type of surgery	Localisation	Size of the lesion (cm^2^)^a^
1	27	M	23.1	DEB	MFC	5.3
2	29	F	29.8	ACI	MFC	3.3
3	42	F	32.4	DEB	NFC	1.9
4	47	F	24.8	DEB	MFC	6.6
5	23	F	19.3	DEB	TROC	1.2
6	48	F	24.7	DEB	MFC	2.2
7	21	M	29.5	MFX	TROC	1.4
8	23	M	23.9	DEB	LFC	1.9
9	27	M	26.6	MFX	LFC	0.4
10	38	M	25.0	DEB	MFC	7.5
11	35	F	28.6	ACI	MFC	4.8
12	47	M	30.0	ACI	MFC	2.2
13	47	M	25.3	MFX	MFC	1.6
14	48	F	27.8	MFX	MFC	1.0
15	50	M	25.7	DEB	LFC	9.0

*M* Male, *F* Female, *DEB* Debridement, *ACI* Autologous chondrocyte implantation, *MFX* Microfracture, *MFC* Medial femoral condyle, *TROC* Trochlea, *LFC* Lateral femoral condyle

^a^Arthroscopic size

### MRI examinations

The MRI examinations were performed in a 3 T MRI unit (Ingenia, Philips Medical Systems, the Netherlands), using a 16-array d-stream transmitter/receiver knee coil. Imaging was performed between January and November 2018. The morphological sequences were performed at the beginning of the examinations. This gave the patients unloading time of about 10 min, before performing T2 mapping sequences. The morphological sequences included a three-dimensional fat-suppressed Proton Density Volume Isotropic Turbo spin echo Acquisition (3D PD VISTA), repetition time / echo time: 1300 / 20 ms, field of view: 140 mm, slice thickness 0.7 mm with isometric voxels and T1-weighted turbo spin echo images in coronal plane, repetition time / echo time: 500 – 700 / 20 ms, field of view: 160 mm, slice thickness 3 mm. T2 relaxation times were obtained from T2 maps derived from two-dimensional, multi-echo spin echo acquisitions performed in sagittal and coronal planes with seven echo times: 13, 26, 39, 52, 65, 78 and 91 ms (repetition time: 4000 ms, field of view: 130 mm, slice thickness 3 mm).

### Image analysis

**Fig. 2:**
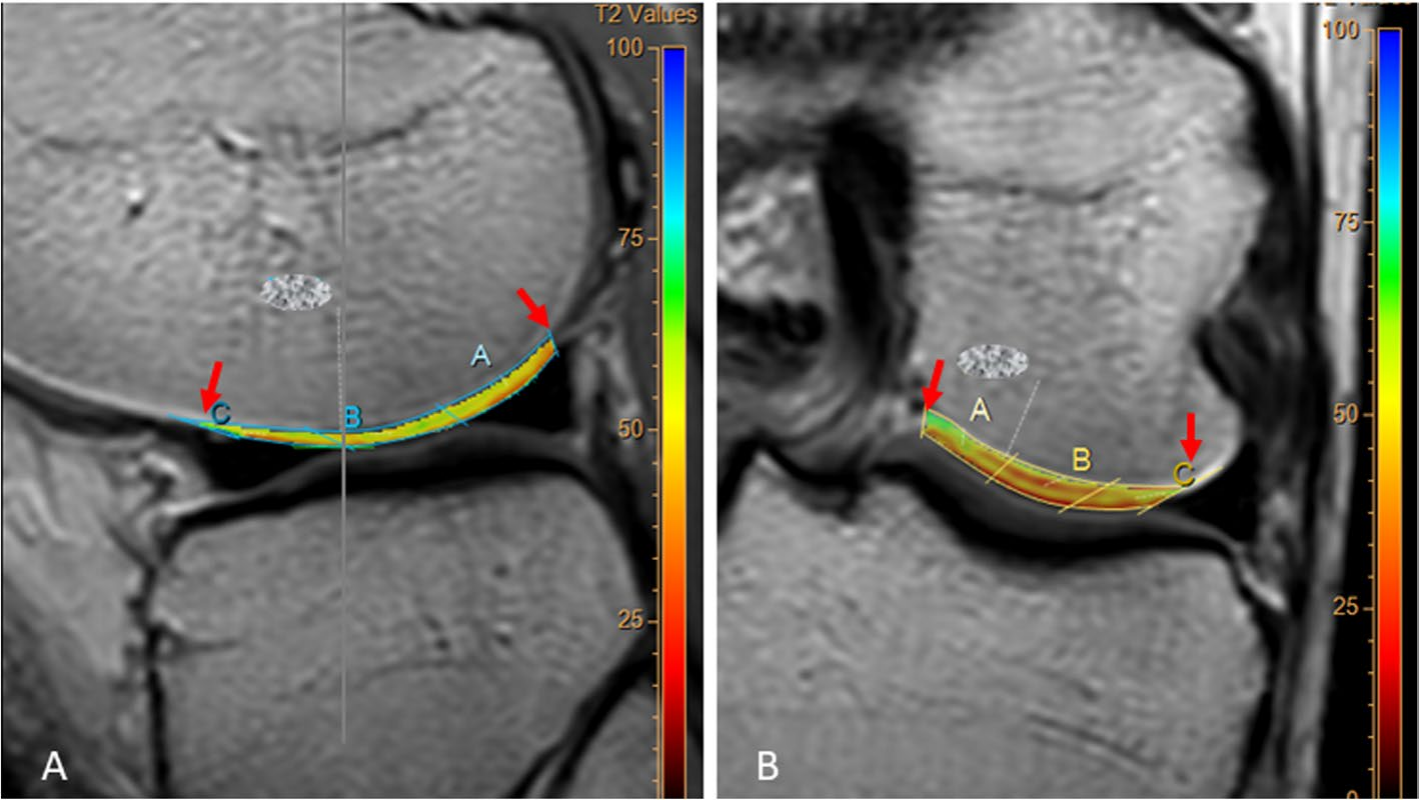
T2 maps of pre-defined ROIs (Regions of Interest) of native cartilage. **a**: (ROI 2) T2 map of the lateral femoral condyle on sagittal plane, between the margins of the posterior and the anterior horns of the meniscus. **b**: (ROI 4) T2 map of the medial femoral condyle on coronal plane at the level with the highest peak of the intercondylar eminence. The arrows indicate the boundaries of the ROIs. The sub-regions **a**, **b** and **c** are automatically generated for each ROI

**Fig. 3:**
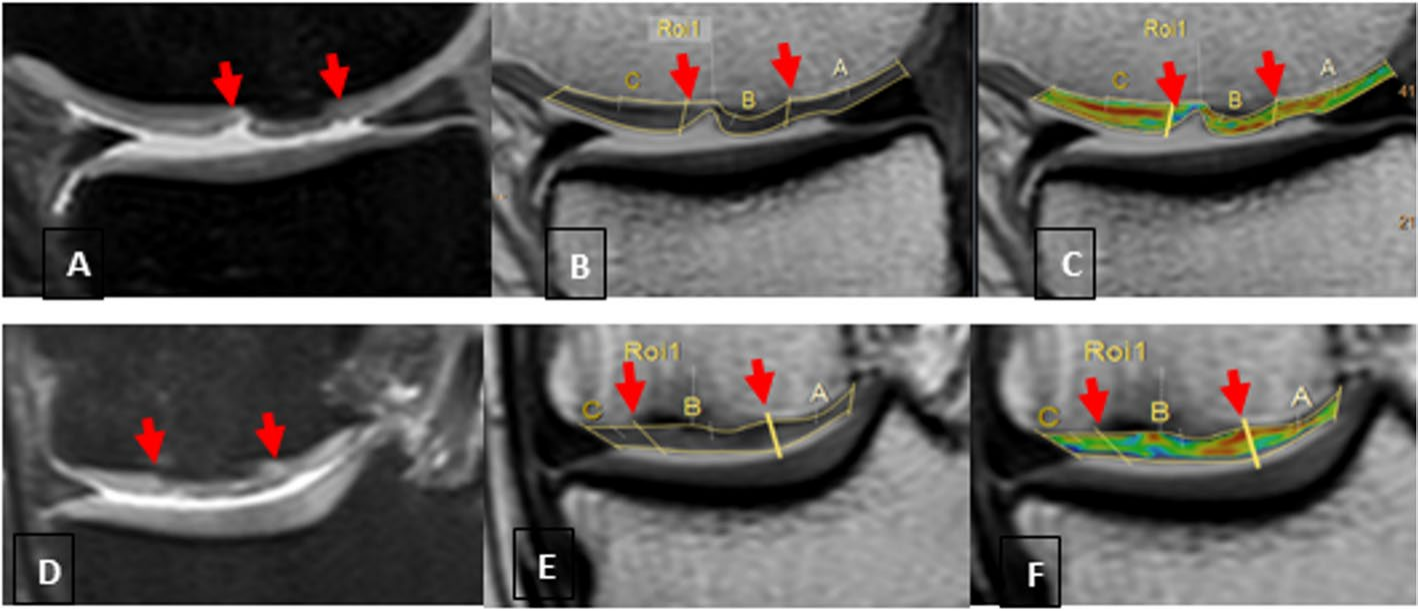
T2 measurements of a treated lesion on medial femoral condyle (images from the same patient). **a** and **d**: Fat suppressed PD VISTA (Proton Density Volume Isotropic Turbo spin echo Acquisition) in sagittal and coronal planes, respectively. Arrows indicate the boundaries of the lesion. **b** and **e**: The same lesion on the first echo of multi-echo sequences as marked by arrows on sagittal and coronal images, respectively. **c** and **f**: Colour maps of the same lesion on sagittal and coronal planes, respectively

To achieve higher reliability and to reflect the everyday practice, four observers from the specialties of radiology and orthopaedics with different levels of experience were chosen. A senior consultant radiologist specialised in musculoskeletal imaging (observer 1), a senior resident of radiology (observer 2) and two consultant orthopaedic surgeons experienced in cartilage surgery (observer 3 and observer 4) evaluated the MRI examinations and measured the T2 relaxation times. All observers were accustomed to evaluate general knee MRI. Observer 1 had ten years of experience in T2 mapping; the other observers did not have any experience in this method. The MRI examinations were anonymised, and the observers were blinded to each other’s ratings and to the type of surgery performed. To enhance the reproducibility of the T2 measurements across the observers, we defined four regions of interest (ROIs) on the weight-bearing articular surfaces of the femur (single-slice measurement for each ROI). We also defined a ROI on the articular surface of the patella (Table 3). We used “MR Cartilage Assessment” application of the Intellispace Portal (Version 10, Phillips Medical Systems, the Netherlands) for T2 measurements. A single-layer approach was applied, since the repair cartilage tissue lacks the typical zonal appearance seen on the native cartilage, and defining layers in repaired lesions may not be reliable [8, 15]. Using MR Cartilage Assessment application, the observers first delineated the interface between the cartilage and the subchondral bone. In the next step, the articular surface of the cartilage was delineated. The software then created three equally large vertical segments in each ROI (sub-regions A, B and C; Fig. 2). The T2 values for each sub-region were calculated automatically by the software. When a treated area extended beyond the boundaries of corresponding sub-region, the observers adjusted the sub-region by moving the boundaries to include the entire treated area (Fig. 3). In cases where the treated area was too large to be included in one sub-region (even by extending the boundaries), the treated area was included in two or three sub-regions. Each sub-region was handled as an independent unit in the statistical calculations. The measurements were repeated by all observers after a minimum of six weeks to assess the intraobserver agreement. This time interval was chosen to preserve the independency of the re-test readings [24]. All observers underwent an instructional course in T2 mapping and using the software held by observer 1 prior to the ratings. An illustrated guide to the T2 mapping software was also provided (available as Additional file), and test readings were conducted on archived MRI scans (different than the study subjects) prior to official rating. Anonymised images of the study subjects were imported into the Intellispace Portal server prior to ratings and the measurements were performed by all observers independently. Since this study was planned as a pure T2 mapping reliability study, the observers did not evaluate any morphological parameters on the MR images.

**Table 3:** Regions of interest for T2 measurements

ROIs on sagittal plane	
ROI 1	The fourth image from the medial border of the medial meniscus, between the margins of the posterior and the anterior horns of the meniscus
ROI 2	The fourth image from the lateral border of the lateral meniscus, between the margins of the posterior and the anterior horns of the meniscus
ROI 3	The mid-sagittal articular surface of the patella
ROIs on coronal plane	
ROI 4	The articular surface of the medial femoral condyle at the level with the highest peak of the intercondylar eminence
ROI 5	The articular surface of the lateral femoral condyle at the level with the highest peak of the intercondylar eminence

*ROI* Region of interest

### Statistical analyses

We used STATA software (StataCorp. 2017. Stata Statistical Software: Release 16. College Station, TX: StataCorp LLC) for statistical calculations and Intraclass Correlation Coefficient (ICC, two-way mixed effect ANOVA) with 95% Confidence Intervals (CI) to calculate the inter- and intraobserver agreement (using the “kappaetc” package). The following scale was used for interpretation of the strength of agreement between the observers [13]:

- ICC ≤ 0.5: poor agreement
- ICC = 0.51 – 0.75: moderate agreement
- ICC ≥ 0.76: good agreement

Two-sample t-test with equal variances was used to compare the mean T2 values on sagittal and coronal planes and the variability of these values was tested using the Levene’s test. P value of ≤ 0.05 was defined as significant. The normality of the data across the observers was assessed by inspection. There were no missing data.

## Results

On ROIs 1 and 2 (sagittal images), three lesions extended through all the three sub-regions and four additional lesions through two sub-regions. On the ROIs 4 and 5 (coronal images), four lesions extended through all three sub-regions and eight additional lesions through two sub-regions. In total, 1131 sub-regional measurements were performed on sagittal images, of which, 360 measurements (32%) included treated lesions. On coronal images, 759 sub-regions were measured, of which, 336 (44%) measurements included treated lesions. Mean T2 values for all ROIs and for sub-regions of repair cartilage tissue (obtained from the first-time measurements of all observers) are presented in Table 4.

**Table 4:** Mean T2 values for different regions

	ROI 1	ROI 2	ROI 3	ROI 4	ROI 5	Sag_ repair	Cor_ repair
Mean	46	51.3	45.1	52.3	50.2	56.0	56.0
SD	6.9	9.3	7.1	7.2	7.4	12.3	13.9

Mean T2 values with corresponding standard deviations (SD) for native and repair tissue. Mean values for each region of interest (ROI) are calculated from the first measurements of all observers and from averaging all 3 sub-regions in each ROI. ROI 1 and 2: sagittal plane, medial and lateral condyles, respectively. ROI 3: mid-sagittal, patella. ROI 4 and 5: coronal plane, medial and lateral condyles, respectively. Sag_repair and Cor_repair: mean sub-regional values for repair tissue on sagittal and coronal images, respectively

### Inter- and intraobserver agreement

We found large variations in the overall interobserver agreement for different regions of native cartilage and repair tissue after surgery, ranging from poor to good (Table 5). For native and repair cartilage tissue, we found good or moderate overall interobserver agreement for the medial femoral condyle on sagittal images (ROI 1) and for the lateral condyle on coronal images (ROI 5). For the other regions, the overall interobserver agreement ranged from poor to good. The poorest agreement was achieved for measurements on repair tissue after ACI. The overall interobserver agreement was moderate or good between the radiologists and poor or moderate between the surgeons (Table 5).

**Table 5:** Interobserver agreement

	**ROI 1**	**ROI 2**	**ROI 3**	**ROI 4**	**ROI 5**
Native^a^	0.76 (0.56 – 0.90)	0.27 (0.11 – 0.46)	0.33 (0.14 – 0.53)	0.46 (0.20 – 0.73)	0.70 (0.57 – 0.82)
ACI^a^	0.51 (0.18 – 0.72)	0.01 (0.00 – 0.39)		0.01 (0.00 – 0.39)	0.84 (0.64 – 0.96)
DEB^a^	0.79 (0.51 – 0.94)	0.23 (0.02 – 0.52)		0.60 (0.24 – 0.87)	0.59 (0.37 – 0.79)
MFX^a^	0.72 (0.38 – 0.95)	0.19 (0.00 – 0.61)		0.31 (0.00 – 0.80)	0.56 (0.24 – 0.86)
Rad	0.90 (0.41 – 0.97)	0.65 (0.41 – 0.81)	0.66 (0.46 – 0.80)	0.78 (0.47 – 0.92)	0.62 (0.38 – 0.79)
Orth	0.66 (0.26 – 0.87)	0.44 (0.14 – 0.67)	0.23 (0.00 – 0.49)	0.40 (0.00 – 0.75)	0.75 (0.57 – 0.86)

Overall interobserver agreement (^**a**^) for T2 measurements of native cartilage, ACI (Autologous Chondrocyte Implantation, DEB (Debridem ent) and MFX (Microfracture)

*ROI* (Region of interest) 1 and 2: sagittal plane, medial and lateral condyles, respectively. ROI 3: mid-sagittal, patella. ROI 4 and 5: coronal plane, medial and lateral condyles, respectively. Interobserver agreement for radiologists (Rad.) and orthopaedic surgeons (Orth.) is presented in the two lower rows

The intraobserver agreement was moderate or good for the radiologists (ICC 0.56 – 0.96), while the agreement for the surgeons varied from poor to good (ICC 0.14 – 0.77) (Table 6).

**Table 6:** Intraobserver agreement

Region	Observer 1	Observer 2	Observer 3	Observer 4
ROI 1	0.95 (0.85 – 0.98)	0.96 (0.88 – 0.99)	0.74 (0.38 – 0.90)	0.43 (0.00 – 0.76)
ROI 2	0.83 (0.71 – 0.90)	0.76 (0.61 – 0.86)	0.58 (0.35—0.75)	0.24 (0.00 – 0.50)
ROI 3	0.76 (0.58 – 0.87)	0.65 (0.42 – 0.81)	0.55 (0.28 – 0.74)	0.25 (0.00 – 0.54)
ROI 4	0.75 (0.41 – 0.91)	0.86 (0.66 – 0.95)	0.17 (0.00 – 0.58)	0.25 (0.00 – 0.66)
ROI 5	0.82 (0.67 – 0.90)	0.81 (0.65 – 0.90)	0.58 (0.32 – 0.76)	0.77 (0.59 – 0.87)
Sag_repair	0.73 (0.55 – 0.84)	0.85 (0.74 – 0.91)	0.50 (0.25 – 0.69)	0.14 (0.00 – 0.41)
Cor_repair	0.56 (0.30 – 0.74)	0.88 (0.79 – 0.93)	0.38 (0.08 – 0.61)	0.29 (0.00 – 0.54)

Intraobserver agreement for T2 measurements of native cartilage and repair cartilage tissue. *ROI* (Region of Interest) 1 and 2: sagittal plane, medial and lateral condyles, respectively. ROI 3: mid-sagittal, patella. ROI 4 and 5: coronal plane, medial and lateral condyles, respectively. Sag_repair and Cor_repair: sub-regions of repair tissue on sagittal and coronal images, respectively

### Differences in T2 values on sagittal and coronal planes

**Fig. 4:**
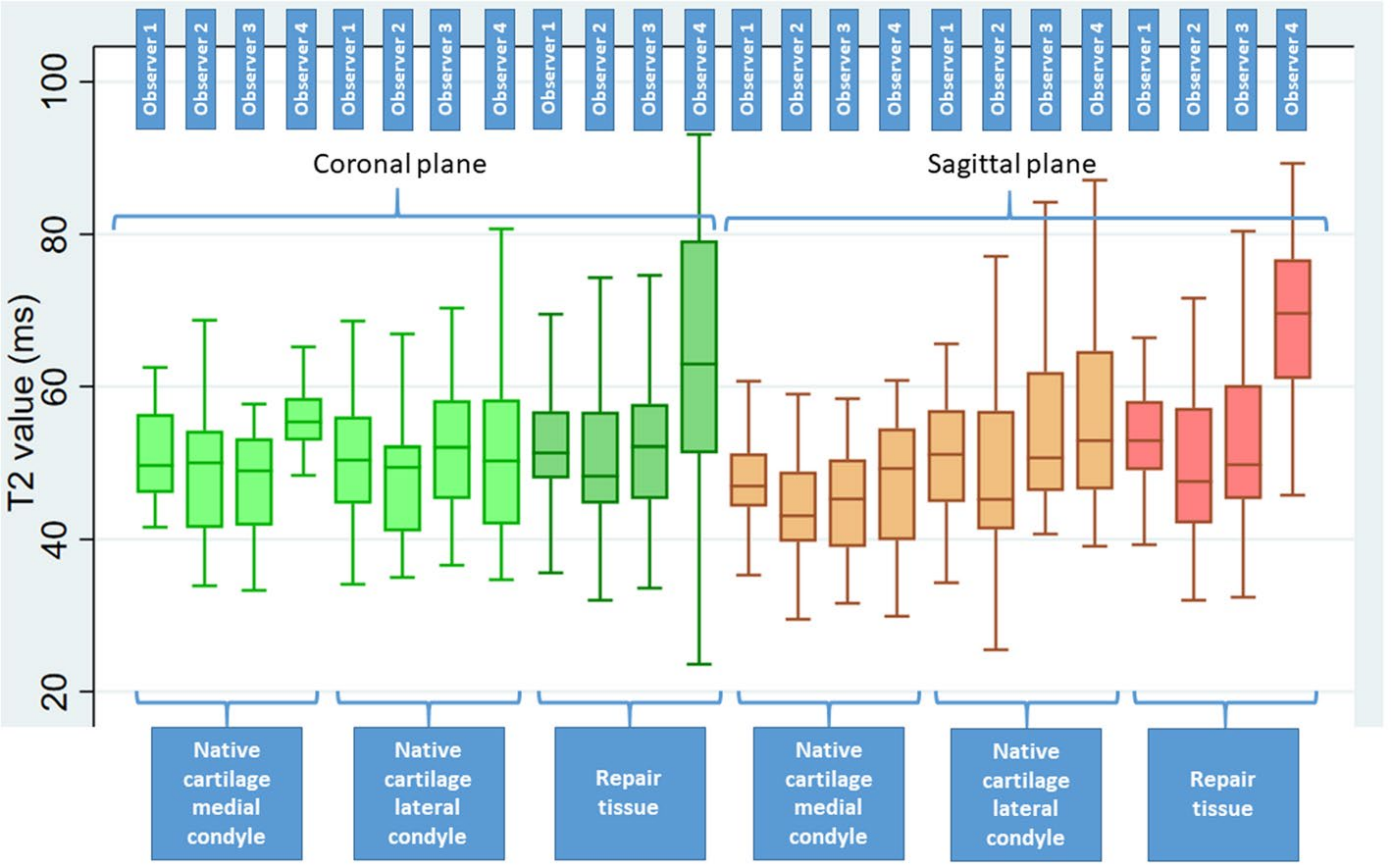
Box plots demonstrating variability of the measured T2 values by the observers in coronal and sagittal planes, for native and repair cartilage tissue

Mean T2 values of the sub-regional measurements (the mean values of the first-time measurements for all observers) for native cartilage of the medial femoral condyle measured on coronal images (ROI 4) were 6.3% higher compared to the measurements on sagittal images (ROI 1, *p* < 0.001). These values were 1.1% higher for the lateral condyle on sagittal images (ROI 2) compared to coronal images (ROI 5, *p* = 0.2). T2 values for repair tissue on the medial condyle were about 10% higher than the native cartilage on sagittal images (*p* < 0.001) and 3.7% higher on coronal images (*P* = 0.9) on the same condyle. On the lateral condyle, the values for repair tissue were 4.7% higher on sagittal images (*p* = 0.001) and 5.7% higher on coronal images compared to native cartilage (*p* < 0.001). Variations in the measurements of T2 values by the four observers (first-time ratings) for native cartilage and repair tissue is demonstrated by box plots in Fig. 4.

## Discussion

The most important findings of the present study were that the image acquisition plane affects the T2 values of the articular cartilage, and that the reliability of these values is influenced by the experience of the observers; the estimated ICC values for inter- and intraobserver agreement suggested that the radiologists in this study demonstrated higher agreement compared to the orthopaedic surgeons. Our results demonstrated that the observer variability was more profound for measurements of the repair cartilage tissue after ACI. There were also differences in the interobserver agreement for the measurements performed on sagittal and coronal images.

The magic angle effect is regarded as a diagnostic pitfall in T2 mapping of articular cartilage. Mosher et al. [17] demonstrated that the greatest changes in T2 values as a consequence of orientation of the fibrils occur in the superficial areas of the cartilage. This difference is caused by more horizontally oriented collagen fibrils in the superficial layer [7, 26]. Differences between the measured T2 values on sagittal and coronal images in our study are likely to be a consequence of the magic angle effect.

A factor that can influence T2 values of repair cartilage tissue measured in different planes is the shape of the lesions, which is irregular and asymmetric. Because of this irregularity, it is impossible to make a perfect imaging plane for the entire lesion, as the defects and irregularities lead to partial volume effects and changes in the fibre orientation. Imaging repair cartilage tissue in at least two planes may reduce the uncertainties related to T2 measurements.

Kurkijärvi et al. [14] evaluated repair cartilage tissue of the distal femur after ACI in coronal and sagittal planes. The authors found higher T2 values for all layers of repair tissue on sagittal plane, but the difference was not statistically significant for the superficial layer on coronal plane. Although the authors could not conclude the exact cause of this difference, they argued that performing T2 mapping sequences in two planes might be necessary due to possible changes of the cartilage adjacent to the repair tissue. Differences in T2 values when measured in different imaging planes, as also demonstrated by our results, suggest that using T2 mapping for evaluation of the results of cartilage repair procedures demands further standardisation. This standardisation may include reproducible measurement methods between observers, for example, making sure that the sagittal and coronal images are perpendicular to the long axis of the cartilage lesion and that measurements are performed in the centre of the treated lesion.

Several publications suggest generally good reproducibility and reliability of T2 mapping as a compositional MRI technique [15, 18, 21]. In a large systematic review and meta-analysis, MacKay et al. [15] evaluated the results of reliability studies of compositional MRI techniques performed on articular cartilage; 36 of the included studies involved T2 mapping. The authors reported interobserver ICCs ranging from 0.17 to 0.99 and intraobserver ICCs ranging from 0.30 to 0.99. The agreement values were lower when the analyses involved small cartilage sub-regions or zonal layers. The review did not reveal any information regarding post-operative cartilage tissue. The quality of the evidence was ranked as moderate by the authors. Lack of information regarding the range of experience and blinding of the observers to the patients’ clinical information were the major limitation of the reliability data. Most of the evaluated publications had only one observer; others had either two observers or the number of the observers was not specified. Six of the evaluated studies had specified the experience of the observer(s), but it was unclear whether the experience was in the related field of T2 mapping. Further, the review did not specify the plane in which the T2 mapping studies were performed.

A major weakness in reporting the experience of the observers of the reliability studies seems to be the definition of “experience”. When it comes to measurement of T2 values, the focus should be the experience of the observers with T2 mapping. This experience may play a key role in demarcating the repair cartilage tissue when evaluating the post-operative results of cartilage repair. As pointed out by MacKay et al., a great portion of the reported studies did not specify whether the observers were blinded to each other and to the clinical data. Strengths of our study included substantial experience of one observer with T2 mapping and blinding of the observers to each other and to the clinical information.

In a study of 25 patients with patellofemoral chondromalacia, van Eck et al. [25] demonstrated excellent inter- and intraobserver agreement for T2 mapping between a musculoskeletal radiologist and a musculoskeletal radiology fellow. In this study, T2 values were measured on native cartilage of the patella on axial images. The patellar cartilage is usually thick and probably easier to demarcate on axial images. Therefore, generalising measurements of T2 values on axial images of the patella to other regions of the knee or to repair tissue may not be reliable.

Our results suggest generally higher T2 values for repair cartilage tissue compared to native cartilage. Welsch et al. [29] demonstrated significant reduction of global mean T2 values of repair tissue after microfracture of the femoral condyle compared to normal cartilage. However, the authors did not find significant differences between mean T2 value of repair cartilage tissue after matrix-associated autologous chondrocyte transplantation and normal cartilage tissue. T2 measurements in the study by Welsch et al. were performed in sagittal plane and the T2 values were assessed in consensus between a musculoskeletal radiologist and an orthopaedic surgeon. In a study by Becher et al.[2], the authors did not find any differences between T2 values of native cartilage and repair tissue after microfracture treatment of cartilage lesions of the talus. The authors reported good interobserver agreement between three independent observers (ICC = 0.8). T2 values of repair tissue in this study were measured by manually drawn ROIs without sub-regions. These values were then compared with the values from a normal looking cartilage selected by the observers. T2 maps were performed in sagittal and coronal planes. However, the authors did not share any information about the differences of T2 values on sagittal and coronal planes.

### Limitations

To increase the reproducibility of the measurements, we used pre-defined ROIs on the articular surfaces. However, separating repair cartilage tissue from native cartilage is challenging and may interfere with the reproducibility of the measurements across the observers. Although the observers in our study could adjust the boundaries of the sub-regions to include the entire treated lesion, inclusion of some normal cartilage in the sub-regions was inevitable. Differences between the observers in adjusting the sub-regions may partly explain the lower agreement values for the repair tissue. Nevertheless, this difference is important to notice, since it indicates that T2 mapping is more challenging and less reliable for repair cartilage tissue than native cartilage. The cartilage adjacent to the repair tissue may not have the same biologic properties as the native cartilage [14], and including this area in the same ROI as the repair tissue may result in more reliable T2 values for the adjacent repair tissue. We suggest that researchers and clinicians perform T2 mapping in at least two different planes and involve radiologists with experience in this method. Further, researchers should be aware of the limitations of T2 mapping when conducting clinical studies with T2 mapping as reference standard.

Another limitation of our study was using a single-slice approach. Choosing one slice of MRI to calculate T2 values of the whole treated area may not be accurate. This limitation is probably more important for larger cartilage lesions, since the chosen slice is less likely to be representable for the whole lesion. Although multislice acquisition for measurement of T2 values has been proved to be clinically applicable, studies suggest that these measurements may not be accurate because of the stimulated echoes and magnetisation transfer affecting the relaxation time of the cartilage [16, 28]. Nevertheless, single-slice approach can potentially limit the generalisability of our results. Limited number of included patients was another limitation of our study. However, with inclusion of sub-regions in sagittal and coronal planes, we achieved reasonable numbers of measurements.

## Conclusion

The results of this study indicate that the image acquisition plane for performing T2 mapping of articular cartilage and the experience of the observers with this method are likely to influence the observer reliability. Researchers who conduct cartilage repair studies with T2 mapping as an endpoint and clinicians who use this method for evaluation of the surgical results should be aware of the limitations of this method.

## Supplementary Information

**Additional file 1.**

## Data Availability

Data generated in this study is available through the corresponding author upon a reasonable request.
