# Supplementary material for: Is T2 mapping reliable in evaluation of native and repair cartilage tissue of the knee?
Source: J Exp Orthop. 2021 Apr 28;8:34. doi: 10.1186/s40634-021-00350-1 (PMC8081777; doi:10.1186/s40634-021-00350-1)
Supplement: Supplementary file 1 — Additional file 1. [file 40634_2021_350_MOESM1_ESM.pdf]

User instructions: MR Cartilage Assessment on Intellispace Portal (ISP, Philips Medical systems) for the NCP reliability study

Is T2 Mapping Reliable in Evaluation of Native and Repair Cartilage Tissue of the Knee?

Journal: Journal of Experimental Orthopaedics

Authors:

Hasan Banitalebi MD[1, 2,] email: hbanitalebi@yahoo.no
Christian Owesen MD PhD[3,4], email: cowesen@gmail.com
Hang Thi Tran MD[1], hngtran@gmail.com
Tor Åge Myklebust PhD5, email: tor.age.myklebust@helse-mr.no
Asbjørn Årøen MD PhD[2 3,4], email: asbjorn.aroen@medisin.uio.no
Per-Henrik Randsborg MD, PhD[3,4,] email: per-henrik.randsborg@ahus.no

1- Department of Diagnostic Imaging, Akershus University Hospital, Norway
2- Institute of Clinical Medicine, University of Oslo, Oslo, Norway
3- Department of Orthopaedic Surgery, Akershus University Hospital, Norway
4- Oslo Sports Trauma Research Centre, Oslo, Norway
5- Department of Research and Innovation, Møre and Romsdal Hospital Trust, Ålesund, Norway

Corresponding author:
Hasan Banitalebi MD, Department of Diagnostic Imaging
Akershus University Hospital
Postbox 1000, 1478 Lørenskog, Norway
Mobile: 0047 971 18 620

By: Hasan Banitalebi MD

Intellispace Portal is a workstation from Philips Medical systems that includes several applications, “MR Cartilage Assessment” is the one we will use in this study. There is a shortcut in the start menu with this symbol: 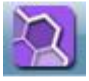

Click on the symbol to start the application, use your credential to log in.

1- The patients in this project can be assessed by writing «MRGE4» under procedure”.

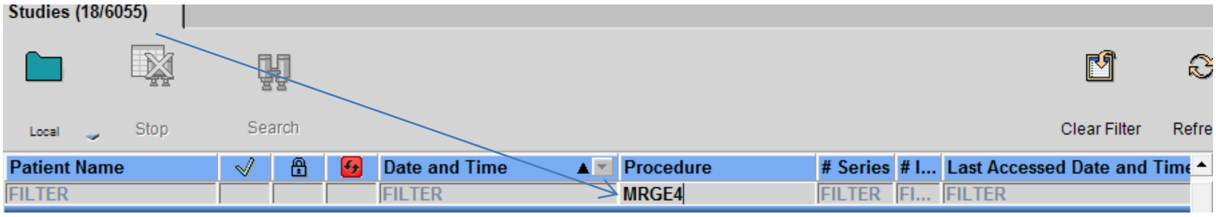

2- The included patients are listed with a unique number and a short name (without social security number):

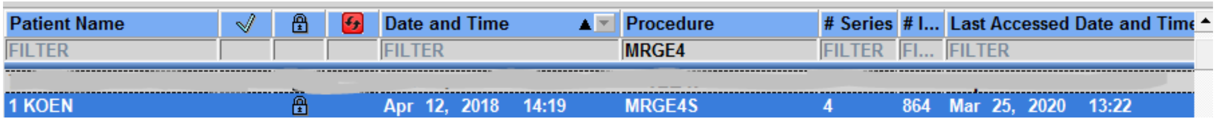

3- There will be three lines under each patient. The first line with large number of images is the 3D sequence (PD VISTA), the other two lines are the calculated multiecho T2 series in sagittal and coronal planes. To start the T2 mapping application, the 3D sequence have to be selected with either the sagittal or the coronal series. Start with the sagittal images.

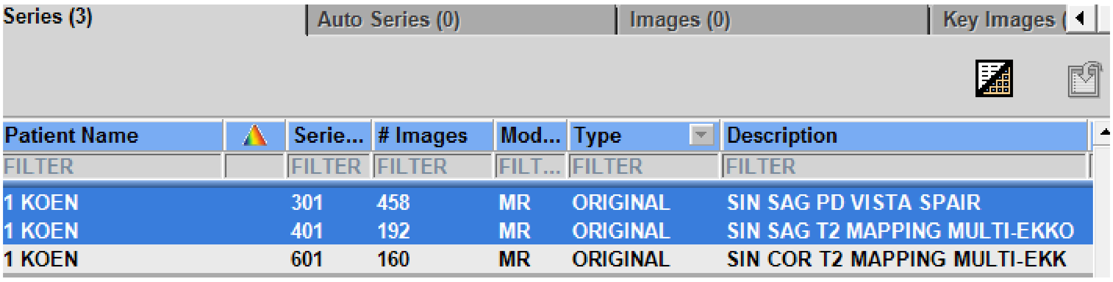

4- Click on the «MR Cartilage assessment» button on the left upper corner:

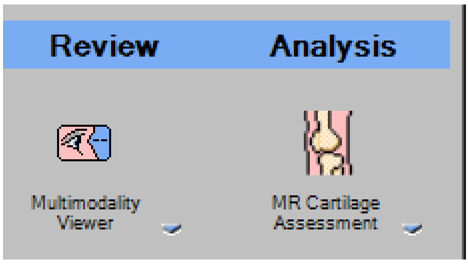

5- Two parallel images will show up. The left one contains the calculated T2 images and the overlay colour map is on the left. Start from the medial side of the knee on sagittal images, find the first image that from the body of the medial meniscus and count four images while moving towards the lateral side; this images should show the anterior and the posterior horns of the meniscus:

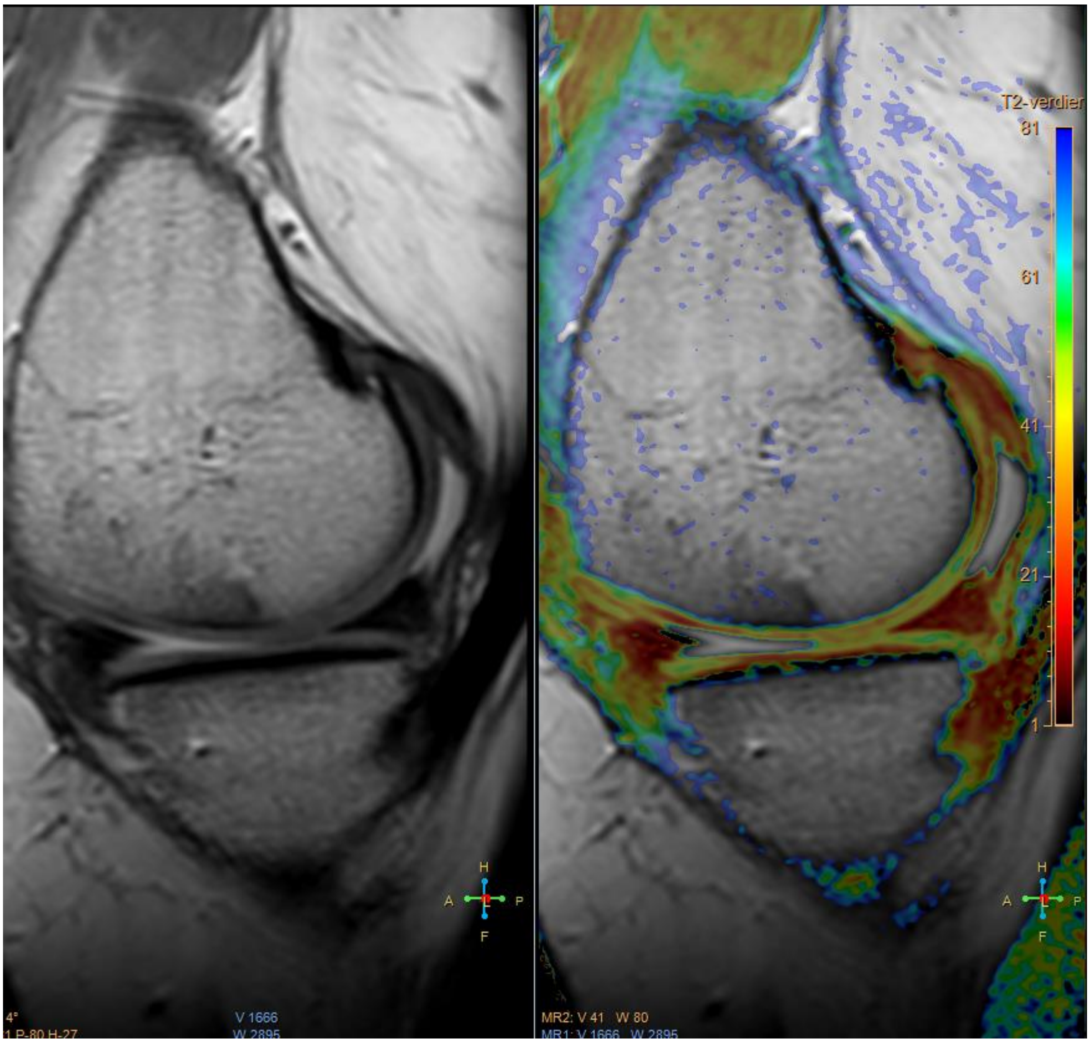

6- From the left panel, choose «Draw bone interface» and demarcate the interface between the subchondral bone and the cartilage. Between the posterior and the anterior boundaries of the meniscal horns:

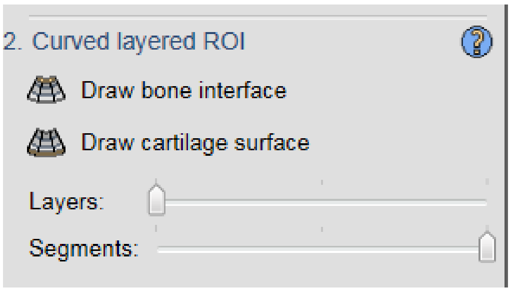

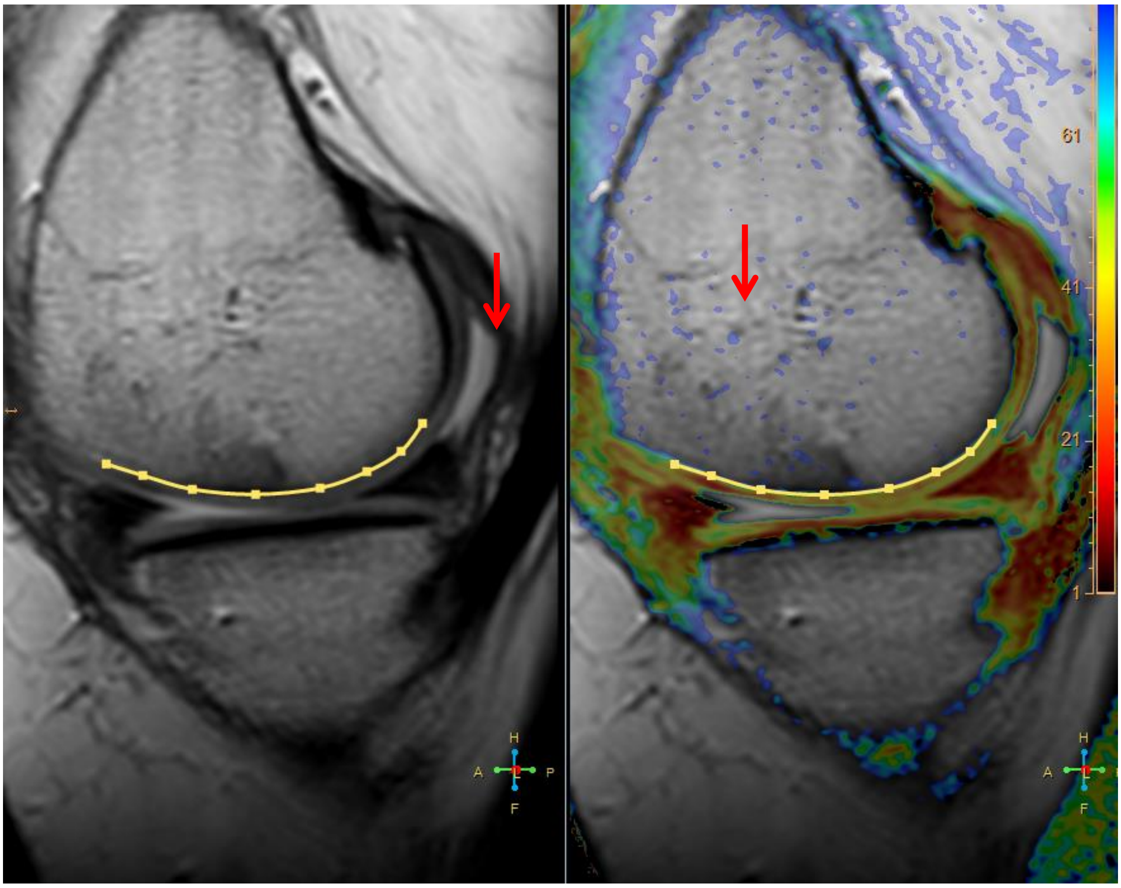

7- Now, choose «Draw cartilage surface», demarcate the cartilage surface by drawing a line and double click when you are done. The software will calculate T2 values for each sub-region

ROI A, B and C). Write down these values for each patient in the provided spread sheet.

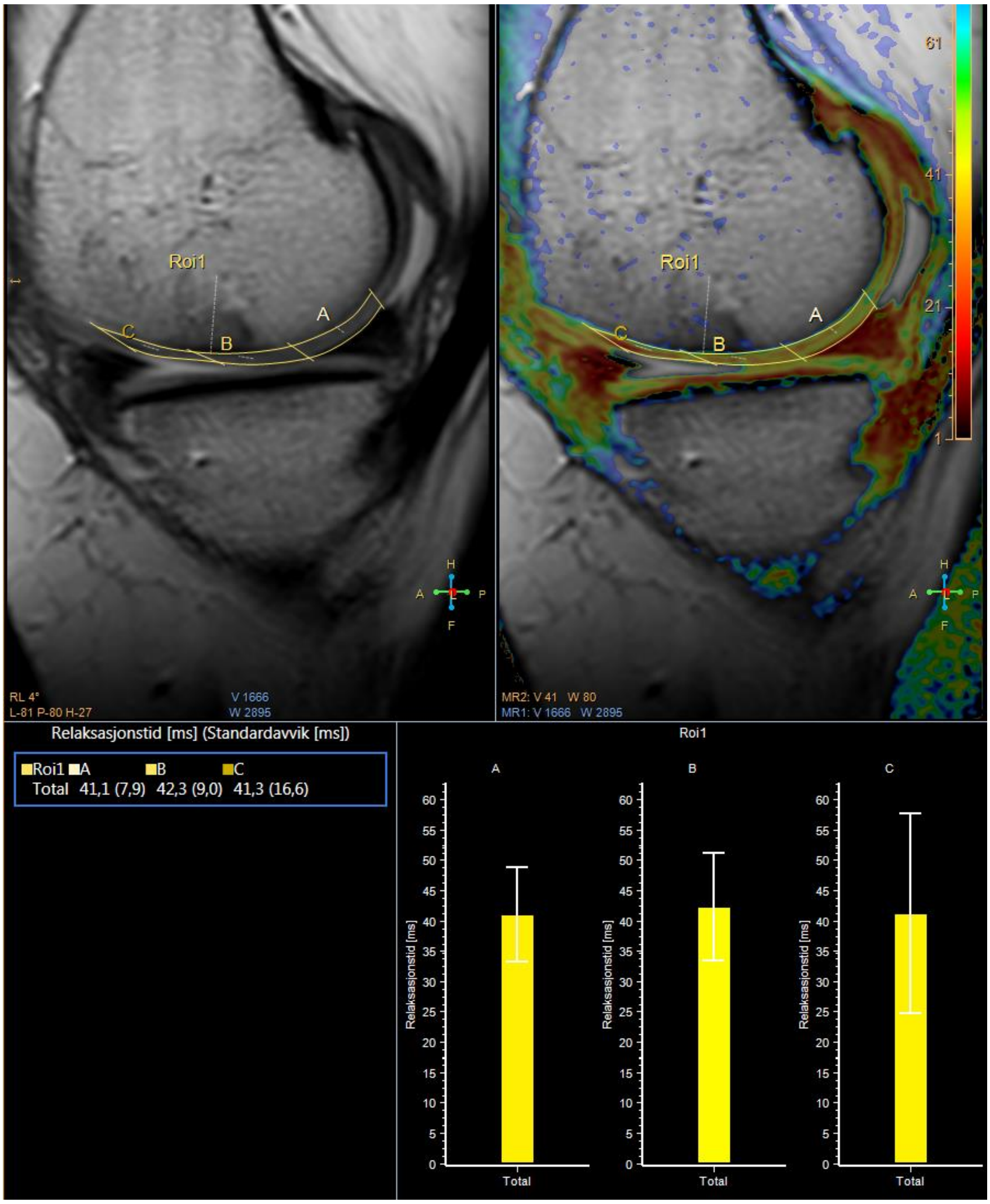

8- Repeat the steps for the Patella mid-sagitally, when you see the whole length of the ACL.

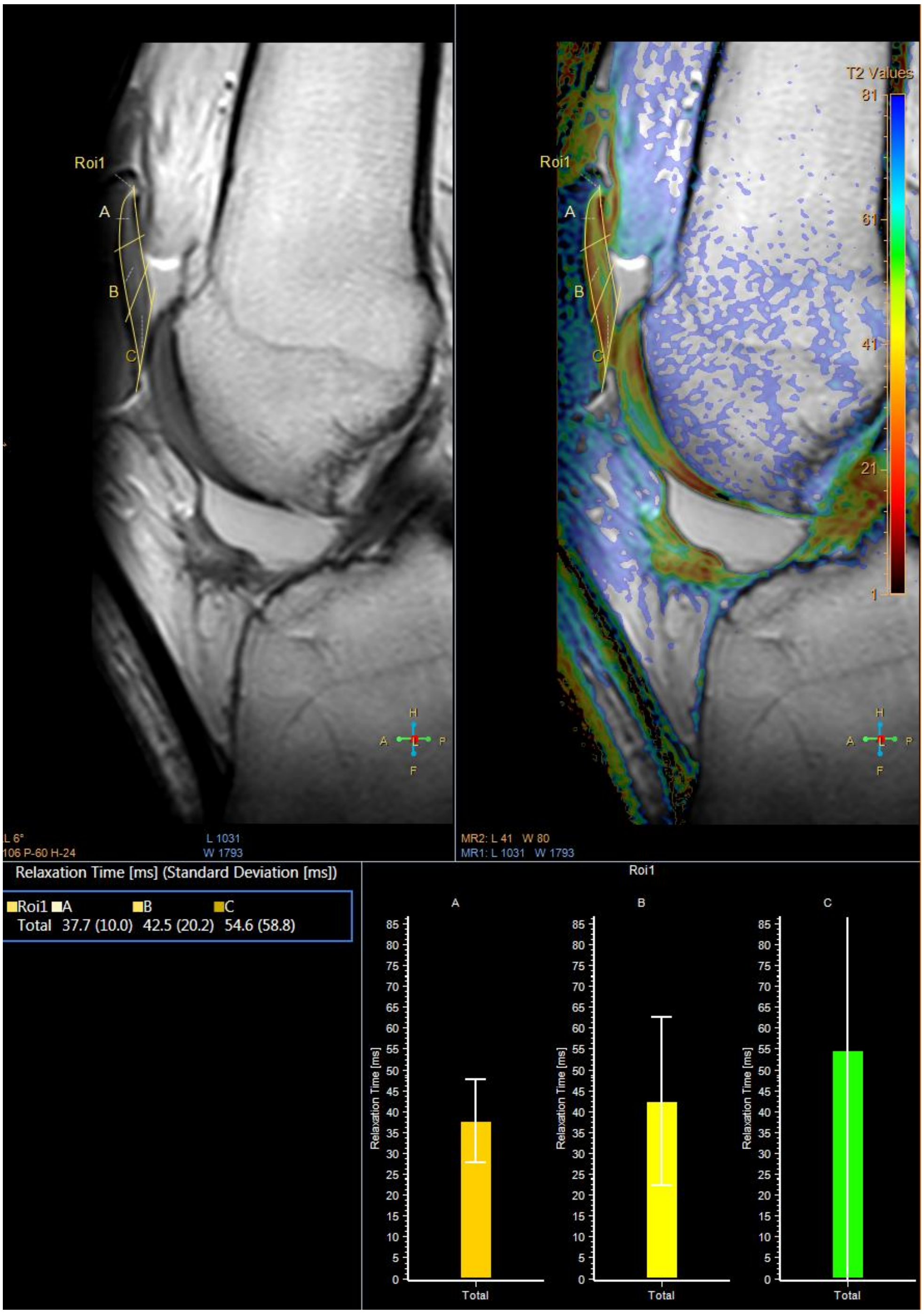

9- Repeat the steps for the lateral condyle, starting from the lateral margin of the meniscal body and performing measurements on the fourth image (between the borders of the posterior and the anterior horns of the meniscus):

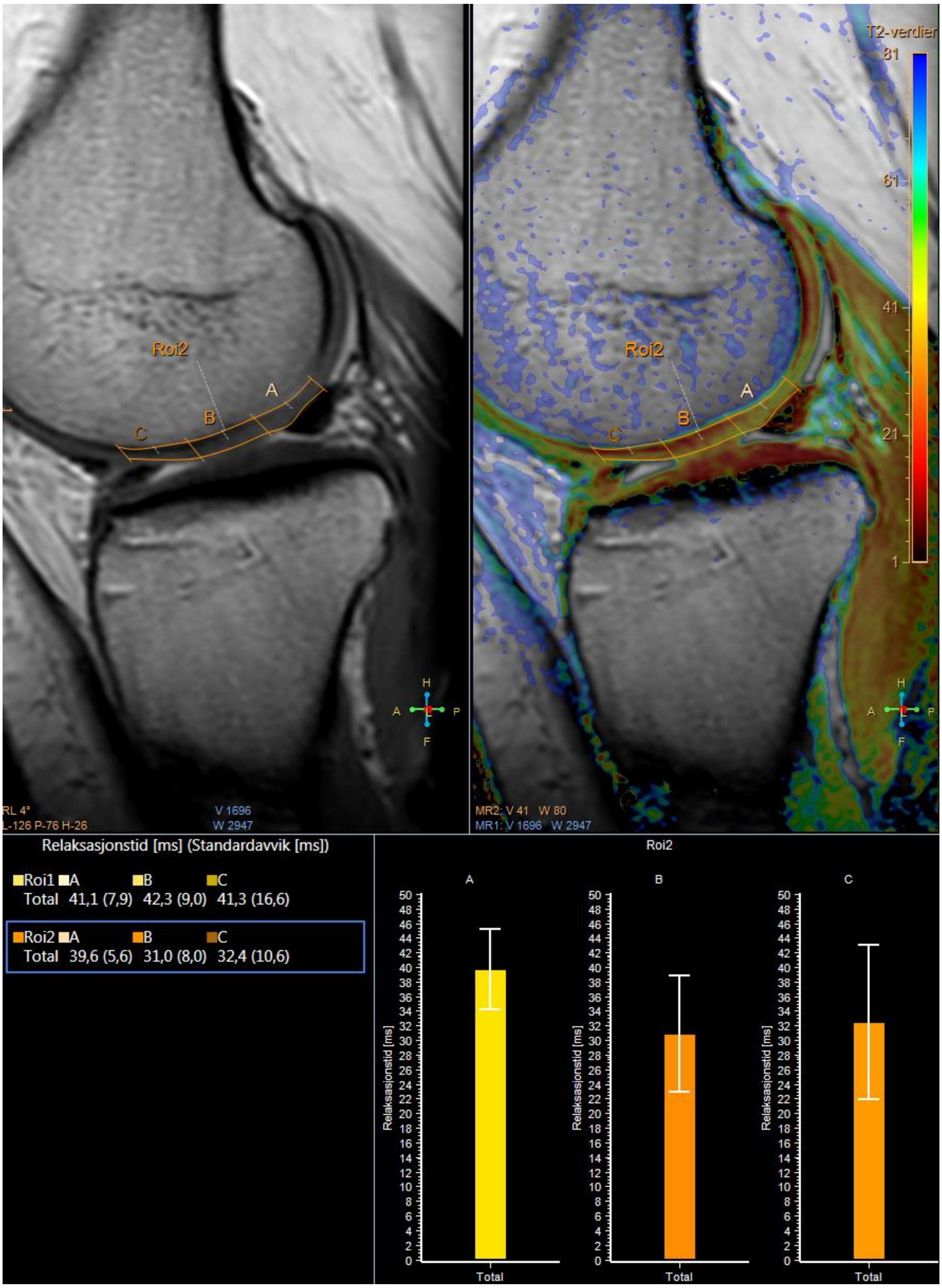

10- Delete all measurements (right click and choose dele measurements)
11- Start measurements on the coronal images by choosing 3D PD VISTA and calculated coronal images and clicking on «MR cartilage assessment». Parallel images with and without colour maps will appear.
12- Find the slice at the level with the highest peak of the intercondylar eminence. Start with the medial femoral condyle. Draw the bone-cartilage interface from centre to medial, draw the cartilage surface from centre to medial and enter the calculated T2 values for each sub-region in the spread sheet.

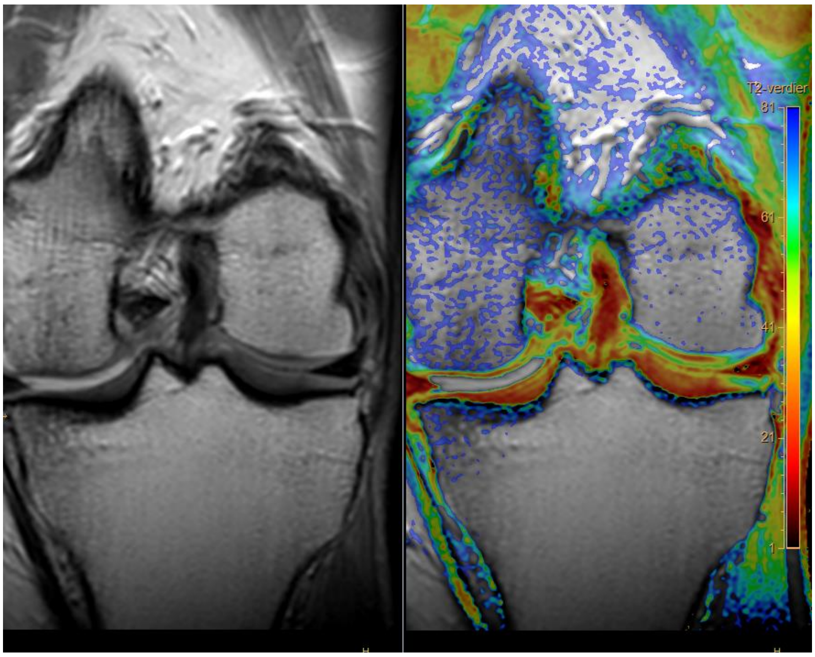

13- Repeat the steps for the lateral condyle, this time by demarcating the cartilage from centre to lateral.

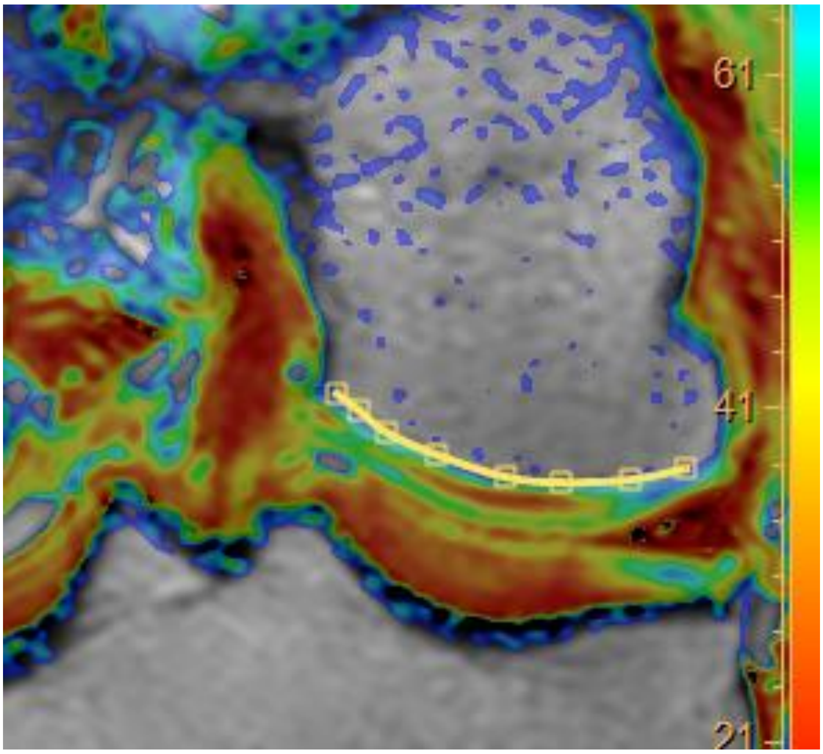

14- Additional notes:

a. When performing measurements on post-operative area, make sure that the entire repaired area is included in the sub-region. This can be done by moving the vertical lines that divide the ROIs into sub-regions. If the post-operative area is too large for one sub-region, try to put it into two or three sub-regions.

b. The intensity of the colours can be adjusted from the left panel:

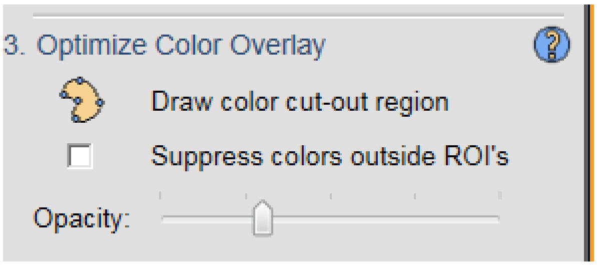

c. Zoom the image until it pans into the window

d. Remember to delete all measurements when you are done with measurements.
